# Systematic review and meta-analysis for prevention of cardiovascular complications using GLP-1 receptor agonists and SGLT-2 inhibitors in obese diabetic patients

**DOI:** 10.1038/s41598-021-89620-7

**Published:** 2021-05-13

**Authors:** Kazushi Uneda, Yuki Kawai, Takayuki Yamada, Sho Kinguchi, Kengo Azushima, Tomohiko Kanaoka, Yoshiyuki Toya, Hiromichi Wakui, Kouichi Tamura

**Affiliations:** 1 Department of Medical Science and Cardiorenal Medicine, Yokohama City University Graduate School of Medicine, 3-9 Fukuura, Kanazawa-ku, Yokohama, 236-0004 Japan; 2 Department of Kampo Medicine, Aizu Medical Center, Fukushima Medical University School of Medicine, Aizuwakamatsu, Japan; 3 Department of Medicine, Mount Sinai Beth Israel, Icahn School of Medicine, Mount Sinai, NY USA

**Keywords:** Diseases, Medical research

## Abstract

Patients with type 2 diabetes mellitus (T2DM) and obesity are at high risk of developing cardiovascular disease (CVD). Both glucagon-like peptide-1 receptor agonists (GLP-1 RAs) and sodium-glucose cotransporter (SGLT-2) inhibitors have been shown to prevent CVD in T2DM patients. Additionally, the two drugs reduce body mass. However, it is unknown which drug is more effective at reducing the risk of CVD in such patients. We searched Medline, EMBASE, and Cochrane Library records to February 20, 2021 and performed a network meta-analysis to compare the efficacy with which the drugs reduced the risk of major adverse cardiovascular events (MACE). We included 102,728 patients in 12 studies containing data of obesity subgroup analyses. In T2DM patients with obesity, GLP-1 RAs significantly reduced the risk of MACE *versus* placebo (relative risk, RR [95% confidence interval, CI]: 0.88 [0.81–0.96]), whereas SGLT-2 inhibitors showed a tendency (RR [95% CI]: 0.91 [0.83–1.00]). In an indirect comparison, GLP-1 RAs were not associated with a significant difference in MACE compared with SGLT-2 inhibitors (RR [95% CI]: 0.97 [0.85–1.09]). Thus, GLP-1 RAs are effective at preventing MACE than placebo in T2DM patients with obesity, although further studies are warranted to conclude their superiority to SGLT-2 inhibitors.

## Introduction

Type 2 diabetes mellitus (T2DM) is an important risk factor for cardiovascular disease (CVD), chronic kidney disease, and mortality. In clinical settings, patients with T2DM often have other cardiometabolic comorbidities, such as hypertension, dyslipidemia, atherosclerotic disease, and obesity^1,2^. Indeed, more than half of patients with T2DM have been reported to be obese^2,3^. Furthermore, because obesity in T2DM increases the risks of CVD and all-cause mortality, the presence of obesity is an important determinant of the prognosis of patients with T2DM^3,4^. The mechanism of this link involves abnormal secretion of adipocytokines, which exacerbates the other CVD risk factors, including hypertension, hyperlipidemia, and insulin resistance^1,5,6^. Therefore, intensive therapy for patients with T2DM and obesity is crucial.

Glucagon-like peptide-1 (GLP-1) receptor agonists (GLP-1 RAs) and sodium-glucose cotransporter (SGLT-2) inhibitors are novel glucose-lowering agents. GLP-1 RAs act as mimetics of the endogenous incretin hormone GLP-1, promote glucose-dependent insulin secretion, and inhibit hepatic glucose production^7^. SGLT-2 inhibitors suppress glucose reabsorption by the renal proximal tubules and exhibit insulin-independent glucose-lowering effects^8^. Moreover, both GLP-1 RAs and SGLT-2 inhibitors have pleiotropic effects that include natriuresis, reductions in blood pressure, and cardiovascular protection^9,10^. Recent randomized controlled studies have shown that GLP-1 RAs and SGLT-2 inhibitors reduce the risks of CVD events^11–14^, and therefore the American Diabetes Association (ADA) recommends the use of GLP-1 RAs and SGLT-2 inhibitors as first-line treatments for patients with T2DM and established or a high risk of atherosclerotic CVD^15^. Furthermore, both GLP-1 RAs and SGLT-2 inhibitors have anti-obesity effects, in contrast to the effects of some other anti-glycemic agents, such as insulin, the use of which tends to be associated with increases in the body mass of patients. Therefore, these two drugs are also recommended for use in overweight patients with T2DM by the ADA^15^. However, no head-to-head trials have been conducted on the efficacy of these drugs for the prevention of CVD in patients with obesity and T2DM. Therefore, we conducted a network meta-analysis to compare the effects of GLP-1 RAs and SGLT-2 inhibitors to prevent CVD in patients with obesity and T2DM.

## Results

### Search results and included studies

**Figure 1:**
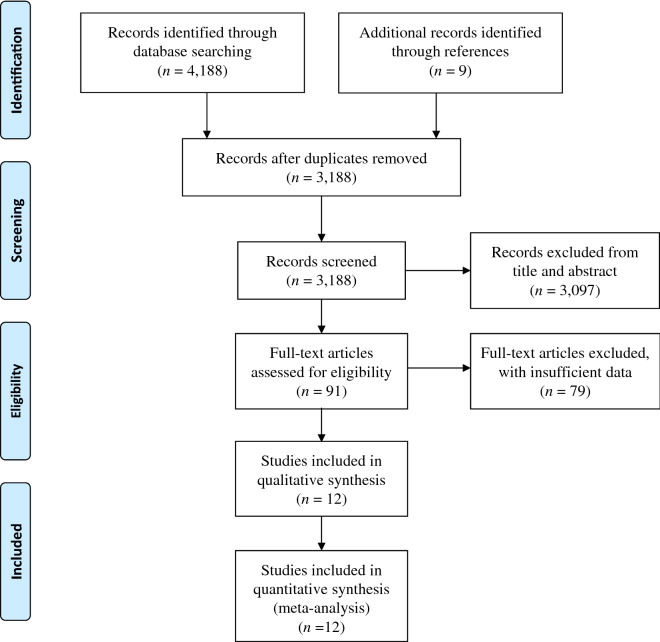
Flow diagram for the study.

Figure 1 shows a flow chart of the study selection procedure. We selected 4,188 studies through database searching and identified a further nine studies through the searches of the reference lists of these articles. After the removal of duplicates, we removed a further 3,097 reports after screening the titles and abstracts, and another 79 because of missing data for upon inspection of the full-text articles. Therefore, 12 studies remained for inclusion in our meta-analysis. Of these, five were placebo-controlled randomized controlled trials (RCTs) of SGLT-2 inhibitors (canagliflozin^16,17^, empagliflozin^13^, ertugliflozin^18^, and dapagliflozin^14^) and seven were placebo-controlled RCTs of GLP-1 RAs (lixisenatide^19^, exenatide^20^, liraglutide^11^, albiglutide^21^, subcutaneous and oral semaglutide^22,23^, and dulaglutide^12^). All the studies, except CREDENCE, set major adverse cardiovascular events (MACE) as the primary endpoint. Eleven studies^11,13,14,16–23^ defined the cut-off value of body mass index (BMI) for obesity as 30 kg/m^2^ and one^12^ defined this cut-off as 32 kg/m^2^.

### Patient characteristics and study quality assessment

The 12 studies included in the meta-analysis are listed in Table 1. In the GLP-1 RA studies the median follow-up time was 15.9–64.8 months and in the studies of SGLT-2 inhibitors it was 31.4–50.4 months. The characteristics of the participants in the included studies are summarized in Table 2. There were 102,728 participants in the included RCTs (55,786 in the GLP-1 RA trials and 46,942 in the SGLT-2 inhibitor trials). In all the studies the mean age of the participants was between 60 and 70, and more than half were male. The mean BMI of the participants was > 30 kg/m^2^ and their mean glycohemoglobin (HbA1c) was 7.4%–8.7%. Their mean systolic and diastolic blood pressures were 130–140 mmHg and 76–80 mmHg, respectively, and the mean circulating low-density lipoprotein-cholesterol concentrations were < 100 mg/dL (2.58 mmol/L) in all the included studies.

**Table 1:** List of included studies.

Study	Study design	Setting	Drug dose (mg/day)	Median follow up (months)	Range of HbA1c (%)	Primary outcome
**GLP-1 RA *vs*. placebo**						
ELIXA	RCT	Multinational	Lixisenatide 20 µg	27.0	5.5–11.0	MACE (including unstable angina)
EXSCEL	RCT	Multinational	Exenatide 2 (weekly)	38.4	6.5–10.0	MACE
LEADER	RCT	Multinational	Liraglutide 1.8	45.6	≥ 7.0	MACE
HARMONY Outcomes	RCT	Multinational	Albiglutide 30–50 (weekly)	19.2	> 7.0	MACE
PIONEER-6	RCT	Multinational	Semaglutide 14 (oral)	15.9	N/A	MACE
REWIND	RCT	Multinational	Dulaglutide 1.5 (weekly)	64.8	≤ 9.5	MACE
SUSTAIN-6	RCT	Multinational	Semaglutide 0.5/1 (weekly)	25.2	≥ 7.0	MACE
**SGLT2i *vs*. placebo**						
EMPA-REG OUTCOME	RCT	Multinational	Empagliflozin 10/25	37.2	7.0–10.0	MACE
CANVAS	RCT	Multinational	Canagliflozin (300/100)	47.1*	7.0–10.5	MACE
CREDENCE	RCT	Multinational	Canagliflozin 100	31.4	6.5–12.0	Renal outcomes
DECLARE-TIMI 58	RCT	Multinational	Dapagliflozin 10	50.4	6.5–12.0	MACE
VERTIS-CV	RCT	Multinational	Ertugliflozin 5/15	36.0	7.0–10.5	MACE

SGLT2i, sodium-glucose cotransporter-2 inhibitor; GLP-1 RA, glucagon-like peptide-1 receptor agonist; RCT, randomized control study; HbA1c, hemoglobin A1c; MACE, major adverse cardiovascular events.

*Follow-up period was mean for CANVAS Program.

**Table 2:** Baseline characteristics of the participants at baseline in the included studies.

Study	Number of participants	Age (years)	Male (%)	BMI (kg/m^2^)	Current smokers (%)	HbA1c (%)	SBP (mmHg)	DBP (mmHg)	eGFR (mL/min/1.73m^2^)	LDL-C (mg/dL)	TG (mg/dL)
**GLP-1 RA *vs*. placebo**											
ELIXA	6,068	60.3	69.3	30.2	11.7	7.7	130	N/A	76.0	78.5	164.5
EXSCEL	14,602	61.9	62.0	32.7	11.7	8.1	135*	80*	78.4	88.0*	N/A
LEADER	9,331	64.3	64.3	32.5	12.1	8.7	136	77	80.4	89.0	N/A
HARMONY Outcomes	9,413	64.1	69.4	32.3	15.7	8.7	135	77	79.0	N/A	N/A
PIONEER-6	3,182	66.0	68.4	32.3	11.0	8.2	136	76	74.0	78.0	N/A
REWIND	9,900	66.2	53.7	32.3	14.2	7.4	137	78	76.9	99.1	141.7
SUSTAIN-6	3,290	64.6	60.7	32.8	N/A	8.7	136	77	N/A	82.3	N/A
Weighted average		63.7	63.2	32.2	12.9	8.2	135	78.0	78.0	88.2	
**SGLT2i *vs*. placebo**											
EMPA-REG OUTCOME	7,020	63.1	71.3	30.6	N/A	8.1	135	77	74.1	85.6	170.6
CANVAS	10,142	63.3	64.2	32.0	17.8	8.2	137	78	76.5	89.0	177.1
CREDENCE	4,392	63.0	66.1	31.3	14.5	8.3	140	78	56.2	96.4	197.9
DECLARE-TIMI 58	17,151	63.9	62.6	32.0	N/A	8.3	135	78	85.2	N/A	N/A
VERTIS-CV	8,237	64.4	70.0	31.9	N/A	8.2	133	76	76.0	89.1	180.6
Weighted average		63.7	65.9	31.7		8.2	136	77	77.3	89.3	179.6

Data are mean values.

SGLT2i, sodium-glucose cotransporter-2 inhibitor; GLP-1 RA, glucagon-like peptide-1 receptor agonist; eGFR, estimated glomerular filtration rate; BMI, body mass index; SBP, systolic blood pressure; DBP, diastolic blood pressure; HbA1c, hemoglobin A1c; LDL-C, low-density lipoprotein-cholesterol; TG, triglyceride; N/A, not available.

*Except for the BP and LDL-C values for EXSCEL (medians).

**Figure 2:**
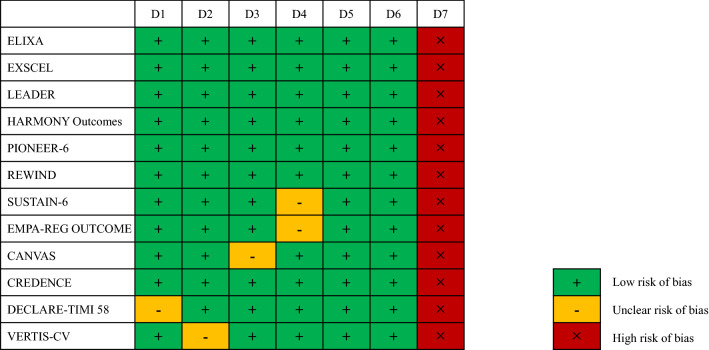
Quality assessment (Cochrane risk of bias tool) for the included RCTs. RCT, randomized control study; domain 1 (D1), random sequence generation; D2, allocation concealment; D3, blinding of participants and personnel; D4, blinding of outcome assessment; D5, incomplete outcome data; D6, selective reporting; D7, other bias.

Figure 2 shows the risks of bias for the included studies. Almost all the included studies were categorized as “low risk.”

### Results of the network meta-analysis

**Figure 3:**
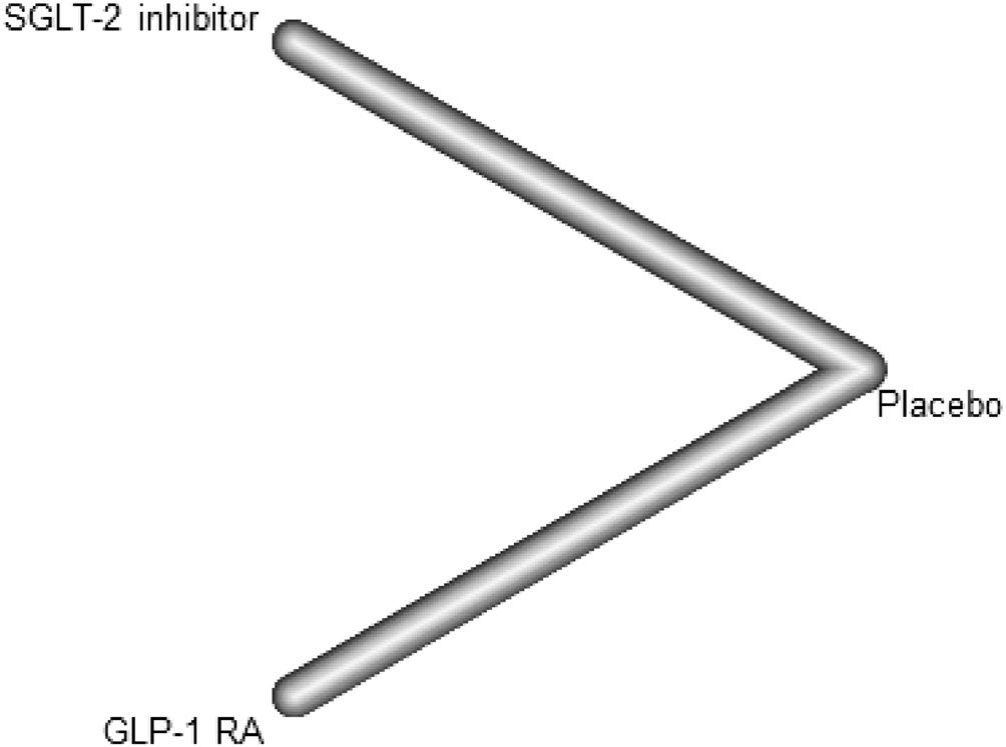
Network plot for the meta-analysis. SGLT-2, sodium-glucose cotransporter 2; GLP-1 RA, glucagon-like peptide-1 receptor agonist.

**Figure 4:**
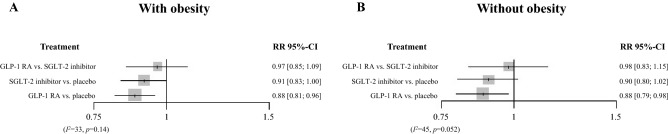
Relative risks of MACE, calculated in the network meta-analysis. Relative risks of MACE in participants (**A**) with obesity and (**B**) without obesity. SGLT-2, sodium-glucose cotransporter 2; GLP-1 RA, glucagon-like peptide-1 receptor agonist; MACE, major adverse cardiovascular events.

Figure 3 shows the network plot. In patients with obesity and T2DM, GLP-1 RAs reduced the risk of MACE *versus* placebo (relative risk, RR [95% confidence interval, CI]: 0.88 [0.81–0.96]). On the other hand, SGLT-2 inhibitors tended to exert cardiac protection compared with placebo (RR [95% CI]: 0.91 [0.83–1.00]) (Fig. 4A). In an indirect comparison, GLP-1 RAs did not show the significant difference in MACE prevention compared with SGLT-2 inhibitors (RR [95% CI]: 0.97 [0.85–1.09]). No significant heterogeneity was found among the studies (*I*^2^ = 33%, *P* = 0.14).

In non-obese participants with T2DM, GLP-1 RAs reduced the risk of MACE *versus* placebo (RR [95% CI]: 0.88 [0.79–0.98]), whereas SGLT-2 inhibitors did not have a statistically significant effect *versus* placebo (RR [95% CI]: 0.90 [0.80–1.02]) (Fig. 4B). However, compared with SGLT-2 inhibitors, GLP-1 RAs did not have a superior effect (RR [95% CI]: 0.98 [0.83–1.15]). Moderate heterogeneity was found among the studies (*I*^2^ = 45%, *P* = 0.052).

### Results of the sensitivity analyses

We conducted sensitivity analyses to validate the initial analysis (Table 3). First, we performed an analysis that excluded the CREDENCE data because the primary outcome of this trial was not MACE, but rather renal outcomes. This analysis showed that GLP-1 RAs significantly reduced the risk of MACE *versus* placebo in participants with T2DM who did or did not have obesity (RR 0.88 [0.82–0.95] and 0.88 [0.79–0.98], respectively), whereas SGLT-2 inhibitors did not (RR 0.94 [0.85–1.03] and 0.91 [0.80–1.04], respectively). However, in T2DM participants with or without obesity, the use of GLP-1 RAs was not associated with a significant reduction in MACE compared with SGLT-2 inhibitors (RR 0.94 [0.83–1.06] and 0.97 [0.81–1.15]). Moderate heterogeneity was found in T2DM with obesity (*I*^2^ = 26%, *P* = 0.20), while significant heterogeneity remained in T2DM without obesity (*I*^2^ = 50%, *P* = 0.037).

**Table 3:** Details of the sensitivity analyses.

Sensitivity analysis	GLP-1 RA (*n*)	SGLT-2 inhibitor (*n*)	Comparison	RR	95% CI	*I*^2^ (%)	*P*-value
**For obese participants with T2DM**							
Without CREDENCE (limiting primary outcomes)	30,760	21,873	GLP-1 RA *vs*. placebo	0.88	0.82–0.95	26	0.20
			SGLT-2 inhibitor *vs*. placebo	0.94	0.85–1.03		
			GLP-1 RA *vs*. SGLT-2 inhibitor	0.94	0.83–1.06		
Without REWIND	26,177	24,239	GLP-1 RA *vs*. placebo	0.89	0.81–0.98	37	0.11
			SGLT-2 inhibitor *vs*. placebo	0.91	0.83–1.00		
			GLP-1 RA *vs*. SGLT-2 inhibitor	0.98	0.86–1.12		
Liraglutide and subcutaneous semaglutide	3,969	24,239	GLP-1 RA *vs*. placebo	0.83	0.70–0.99	42	0.13
			SGLT-2 inhibitor *vs*. placebo	0.91	0.82–1.01		
			GLP-1 RA *vs*. SGLT-2 inhibitor	0.91	0.75–1.11		
Daily GLP-1 RA	9,028	24,239	GLP-1 RA vs. placebo	0.93	0.78–1.10	55	0.038
			SGLT-2 inhibitor *vs*. placebo	0.91	0.81–1.02		
			GLP-1 RA *vs*. SGLT-2 inhibitor	1.02	0.83–1.25		
Weekly GLP-1 RA	21,732	24,239	GLP-1 RA *vs*. placebo	0.87	0.78–0.95	30	0.19
			SGLT-2 inhibitor *vs*. placebo	0.91	0.84–1.00		
			GLP-1 RA *vs*. SGLT-2 inhibitor	0.95	0.83–1.08		
**For non-obese participants with T2DM**							
Without CREDENCE (limiting primary outcomes)	21,992	15,591	GLP-1 RA *vs*. placebo	0.88	0.79–0.98	50	0.037
			SGLT-2 inhibitor *vs*. placebo	0.91	0.80–1.04		
			GLP-1 RA *vs*. SGLT-2 inhibitor	0.97	0.81–1.15		
Without REWIND	16,675	17,617	GLP-1 RA *vs*. placebo	0.86	0.76–0.98	49	0.040
			SGLT-2 inhibitor *vs*. placebo	0.90	0.79–1.02		
			GLP-1 RA *vs*. SGLT-2 inhibitor	0.93	0.75–1.16		
Liraglutide and subcutaneous semaglutide	2,339	17,617	GLP-1 RA *vs*. placebo	0.84	0.66–1.07	56	0.047
			SGLT-2 inhibitor *vs*. placebo	0.90	0.78–1.03		
			GLP-1 RA *vs*. SGLT-2 inhibitor	0.94	0.71–1.24		
Daily GLP-1 RA	6,519	17,617	GLP-1 RA *vs*. placebo	0.94	0.81–1.09	29	0.21
			SGLT-2 inhibitor *vs*. placebo	0.90	0.81–1.01		
			GLP-1 RA *vs*. SGLT-2 inhibitor	1.04	0.87–1.25		
Weekly GLP-1 RA	15,473	17,617	GLP-1 RA *vs*. placebo	0.85	0.73–0.98	53	0.039
			SGLT-2 inhibitor *vs*. placebo	0.90	0.79–1.03		
			GLP-1 RA *vs*. SGLT-2 inhibitor	0.94	0.77–1.14		

SGLT2i, sodium-glucose cotransporter-2 inhibitor; GLP-1 RA, glucagon-like peptide-1 receptor agonist; RR, relative risk; CI, confidence interval.

Next, we conducted an analysis in which we excluded the REWIND data because in this trial obesity was defined using a BMI ≥ 32 kg/m^2^. For participants with obesity and T2DM, GLP-1 RA administration was associated with a lower incidence of MACE (RR 0.89 [0.81–0.98]), whereas SGLT-2 inhibitor administration showed a trend (RR 0.91 [0.83–1.00]). However, no significant difference was observed between the use of GLP-1 RAs and SGLT-2 inhibitors with respect to cardiovascular protection (RR 0.98 [0.86–1.12]). There was moderate heterogeneity among the studies (*I*^2^ = 37%, *P* = 0.11). Similar results were obtained for non-obese participants with T2DM (GLP-1 RAs *vs*. placebo: RR 0.86 [0.76–0.98]; SGLT-2 inhibitors *vs*. placebo: RR 0.90 [0.79–1.02]; GLP-1 RAs *vs*. SGLT-2 inhibitors: RR 0.93 [0.75–1.16]), but there was significant heterogeneity among the studies (*I*^2^ = 49%, *P* = 0.040).

Third, we assessed the cardiac protective effects by liraglutide and subcutaneous semaglutide, that had been reported to reduce patients’ body weight in double-blind RCTs^24,25^. For participants with T2DM and obesity, there was no significant difference in the indirect comparison of GLP-1 RAs and SGLT-2 inhibitors (RR [95% CI]: 0.91 [0.75–1.11]). However, GLP-1 RAs significantly inhibited MACE (RR [95% CI]: 0.83 [0.70–0.99]), although SGLT-2 inhibitors did not reach the statistical difference (RR [95% CI]: 0.91 [0.82–1.01]). There was moderate heterogeneity among the studies (*I*^2^ = 42%, *P* = 0.13).

Lastly, we focused on the frequency of administration of GLP-1RAs. In the participants with obesity and T2DM, weekly GLP-1 RAs (exenatide, dulaglutide, albiglutide, and subcutaneous semaglutide) reduced the risk of MACE *versus* placebo (RR 0.87 [0.78–0.95]), whereas daily GLP-1 RAs (lixisenatide, liraglutide, and oral semaglutide) did not (RR 0.93 [0.78–1.10]). There were moderate to high heterogeneity among the studies in both analyses (*I*^2^ = 30% and 55%, respectively). An indirect comparison of GLP-1 RAs and SGLT-2 inhibitors, administered daily or weekly, did not reveal significant differences in the risks of MACE in T2DM patients with obesity.

## Discussion

We have described the first network meta-analysis to compare the use of GLP-1 RAs and SGLT-2 inhibitors to reduce cardiovascular risk in the presence and absence of obesity. We found that GLP-1 RAs are superior to placebo for the prevention of MACE in T2DM patients with and without obesity, whereas SGLT-2 inhibitors show a tendency but do not outperform placebo in T2DM patients with obesity. However, in T2DM patients with or without obesity, there is no significance in the risk reduction of MACE between GLP-1 RAs and SGLT-2 inhibitors. Sensitivity analyses generated similar findings, which supports the validity of our findings.

Recent meta-analyses have shown protective effects of GLP-1 RAs and SGLT-2 inhibitors against CVD in patients with T2DM^26–28^. However, no head-to-head trials have compared the use of these drugs for the prevention of MACE in patients with obesity and T2DM. In the present study, we cannot conclude the superiority between GLP-1 RAs and SGLT-2 inhibitors in the risk reduction of MACE in T2DM with obesity (Fig. 4). Previous meta-analysis has showed that GLP-1 RAs and SGLT-2 inhibitors may have different effects on each component of three-point MACE, which comprised cardiovascular death, myocardial infarction, and stroke^29^. Particularly, a recent network meta-analysis has reported that GLP-1 RAs inhibited the risk of stroke than SGLT-2 inhibitors, although the risk of cardiovascular death and myocardial infarction is comparable^30^. These factors may influence on our results because our study assessed these diseases together.

The present data indicate that GLP-1 RAs reduce the risk of MACE compared with placebo in T2DM patients with obesity (Fig. 4). Furthermore, the sensitivity analysis that was performed after the exclusion of the CREDENCE data revealed a significant reduction in the risk of cardiovascular events compared with placebo in patients taking GLP-1 RAs with obesity (Table 3). In obesity, greater secretion of adipocytokines by visceral fatty tissues worsens T2DM and CVD through the induction of insulin resistance, endothelial dysfunction, and hypercoagulability^5^. Therefore, we hypothesize that GLP-1 RAs reduce the incidence of CVD events in obese T2DM patients by reducing the activation of adipocytokine pathways. Notably, some RCTs have shown that exenatide, one of the GLP-1 RAs, reduces the intra-abdominal fat volume, adipocytokine concentrations, inflammation, and insulin resistance in obese patients with T2DM^31^. Furthermore, GLP-1 RAs have anti-obesity effects through the regulation of appetite via central GLP-1 receptors, which may also have contributed to the present findings^4,15^. In our sensitivity analyses, liraglutide and subcutaneous semaglutide, drugs reported to reduce patients' body weight in large RCTs^24,25^, also show the risk reduction of MACE compared with placebo for obese T2DM participants (Table 3). However, further research is needed to confirm the mechanisms whereby GLP-1RAs protect against CVD in patients with obesity and T2DM.

We did not identify a significant advantage of SGLT-2 inhibitors over placebo, although they showed a tendency in obese T2DM. To our knowledge, no previous trial has assessed the efficacy of SGLT-2 inhibitors with respect to a reduction in the risk of MACE in patients who were or were not obese. However, some previous reviews have identified anti-obesity effects of SGLT-2 inhibitors^32,33^. Among the studies included in the present meta-analysis, only canagliflozin caused a significant reduction in the incidence of MACE in the obesity sub-group of CREDENCE and CANVAS, while other SGLT-2 inhibitors did not^13,14,16–18^, and hypotheses have been proposed to explain this inconsistency in the effects of SGLT-2 inhibitors on the incidence of MACE^34^. In particular, SGLT-2 inhibitors differ in their selectivity for SGLT-2 and SGLT-1^35^. SGLT-1 is expressed in the renal proximal tubules, but distally to SGLT-2^8,36^. Studies of animal models of diabetes mellitus have shown that renal SGLT-2 and SGLT-1 expression is upregulated and glucose reabsorption is greater^37,38^. Because some previous studies have shown that renal SGLT-1 activation reduces urinary glucose excretion, SGLT-2 inhibitors with low selectivity, and therefore a relatively high affinity for SGLT-1, may be more effective at reducing cardiometabolic risk than those with high selectivity^33,38,39^. A previous network meta-analysis showed that the use of canagliflozin is associated with better improvements in cardiometabolic markers in T2DM patients than that of other SGLT-2 inhibitors^40^. In the present study, the fact that the SGLT-2/1 selectivity of canagliflozin is lower than that of other SGLT-2 inhibitors might have affected the results, especially in patients with obesity and T2DM^32,35,41^.

Since GLP-1 RAs and SGLT-2 inhibitors have different pharmacological targets, the combination therapy of the two drugs is expected to exert various benefits. In fact, recent studies have suggested that the combination therapy of the two drugs could have the better effects on body weight, metabolic parameters, and cardiovascular function in T2DM patients than monotherapy^42,43^. Moreover, SGLT-2 inhibitors have established evidence reducing the risk of worsening heart failure in patients with low ejection fraction regardless of T2DM^44^. Further investigations are warranted to clarify the cardiac protective effects of SGLT-2 inhibitors and how to select or combine the two drugs depending on the patients’ CVD risks.

The present study had some limitations. First, we could not conduct sub-analyses with respect to factors affecting BMI, including age, sex, and ethnicity. Second, we observed heterogeneity in some of the sensitivity analyses. Third, we were not able to assess the change in the body mass of the participants in each RCT. Fourth, splitting each trial's data into obese and non-obese subgroups reduced the power and therefore reduced the likelihood of demonstrating a benefit.

In conclusion, GLP-1 RAs reduce the risk of MACE *versus* placebo in patients with T2DM and obesity, whereas SGLT-2 inhibitors tend to be statistically favorable but do not show a significant difference. Further studies are warranted to conclude GLP-1 RAs’ superiority of cardiovascular protection to SGLT-2 inhibitors in T2DM patients with obesity.

## Methods

### Data sources

The present network meta-analysis was performed according to the PRISMA (Preferred Reporting Items for Systematic Reviews and Meta-Analyses) extension statement for network meta-analysis^45,46^ and registered with PROSPERO (ID:CRD42021245663). We searched electronic databases of EMBASE, Medline, and the Cochrane Library on February 20, 2021. We used the following keywords: (“GLP-1” OR “glucagon-like peptide-1 receptor” [MeSH] OR “glucagon-like peptide-1 receptor agonist” OR “lixisenatide” OR “liraglutide” OR “semaglutide” OR “albiglutide” OR “dulaglutide” OR “exenatide”) OR (“SGLT-2” OR “sodium-glucose cotransporter-2 inhibitor” [MeSH] OR “SGLT-2 inhibitor” OR “empagliflozin” OR “canagliflozin” OR “luseogliflozin” OR “dapagliflozin” OR “ertugliflozin” OR “tofogliflozin” OR “sergliflozin” OR “remogliflozin” OR “ipragliflozin”) AND (“diabetes mellitus type 2” OR “diabetes mellitus, type 2” [MeSH] OR “type 2 diabetes mellitus” OR “diabetes” OR “diabetes mellitus” OR “T2D”) AND (“coronary artery disease” [MeSH] OR “myocardial infarction” [MeSH] OR “cerebrovascular disorders” [MeSH] OR “cardiovascular disease” [MeSH] OR “heart failure” [MeSH] OR “cardiovascular outcome” OR “CVA” OR “stroke” [MeSH] OR “cerebrovascular accident” OR “major adverse cardiovascular event” OR “MACE” OR “major adverse cardiac event” OR “cardiac event” OR “mortality” [MeSH] OR “cardiovascular mortality” OR “death”) AND (“random” OR “trial” OR “placebo”). Additionally, we screened the reference lists of articles reporting meta-analyses to identify further relevant studies.

### Selection of studies

Two independent reviewers (KU and YK) blindly checked the titles and abstracts of the identified articles. Next, the eligibility of studies with respect to the inclusion and exclusion criteria was judged by the two authors. If there was disagreement, a third senior reviewer (TY) was consulted and the matter discussed until a consensus was achieved.

The inclusion criteria were as follows: (i) RCTs published as peer-reviewed articles; (ii) all participants were ≥ 18 years of age and had T2DM; (iii) comparison of treatment (GLP-1 RA or SGLT-2 inhibitor) with placebo; (iv) comparison of the risk of MACE between the two groups; and (v) MACE data recorded according to BMI. The exclusion criteria were as follows: (vi) experimental animal study and (vii) insufficient data to evaluate the RRs of MACE even after contacting the authors.

### Data extraction and assessment of bias

After selecting the studies, the two independent reviewers (KU and YK) extracted data regarding the onset of MACE in participants that underwent treatment with either class of drug or placebo groups from each report. A third senior reviewer (TY) was responsible for resolving any anomalies regarding the data or quality assessment. To identify bias in the RCTs, we used the Cochrane risk of bias assessment^47^.

### Outcomes

The primary endpoint was three-point MACE, which comprised cardiovascular death, myocardial infarction, and stroke.

### Statistical analysis

We used the "netmeta" package (version 1.1–0) and the R programming language (R Foundation for Statistical Computing, Vienna, Austria). To calculate the RRs and 95% CIs, we used the Mantel–Haenszel method. A random-effects model was used for the analysis. Study heterogeneity was evaluated using the probability value of the *I*^2^ variable, and was graded as low, moderate, or high if *I*^2^ was 25%, 50%, or 75%, respectively.

## Data Availability

The datasets analyzed during the current study are available from the corresponding author on reasonable request.
